# Survival of HIV/HCV co-infected patients before introduction of HCV direct acting antivirals (DAA)

**DOI:** 10.1038/s41598-019-48756-3

**Published:** 2019-08-29

**Authors:** L. Dold, C. Schwarze-Zander, C. Boesecke, R. Mohr, B. Langhans, J.-C. Wasmuth, C. P. Strassburg, J. K. Rockstroh, U. Spengler

**Affiliations:** 1 Department of Internal Medicine I, Rheinische Friedrich-Wilhelms University Bonn, Bonn, Germany; 2 German Center for Infection Research (DZIF), partner site Cologne-Bonn, Bonn, Germany

**Keywords:** Infectious diseases, Liver diseases, Viral hepatitis

## Abstract

HIV/HCV infection is supposed to substantially reduce survival as compared to HIV mono-infection. Here, we compared longtime-survival and causes of death in a cohort of HIV- and HIV/HCV-co-infected patients on combined antiretroviral therapy (cART), before introduction of HCV direct acting antivirals (DAA). 322 Caucasian patients with HIV (n = 176) and HIV/HCV-infection (n = 146) were enrolled into this study. All patients were recruited between 2003 and 2004 and followed until 01.01.2014. We compared overall survival between the two groups by the Kaplan-Meyer method and identified independent factors associated with long-time survival by conditional Cox regression analysis. In total 46 (14.3%) patients died during the observation period (HIV infection: n = 23 (13.1%), HIV/HCV infection: n = 23 (15.8%) but overall-survival did not differ significantly between HIV/HCV-infected and HIV mono-infected patients (p = 0.619). Survival was substantially better in patients with complete suppression of HIV replication below the level of detection than in those with residual viremia (p = 0.001). Age (p = 0.008), γ-glutamyltranspeptidase (p < 0.0001) and bilirubin (p = 0.008) were significant predictors of survival irrespective from HCV co-infection. Complete repression of HIV replication on cART is the key factor determining survival both in HIV- and HIV/HCV-co-infected patients, while HCV co-infection and therapy without DAAs seem to affect survival to a lesser extent. Thus, patients with HIV/HCV co-infection require particularly intensive cART.

## Introduction

Hepatitis C (HCV) co-infection occurs frequently in human immunodeficiency virus (HIV)-infected patients since HCV and HIV share the same routes of transmission^1^. Consequently, certain risk groups, e.g. hemophiliacs, patients with intravenous drug abuse (IVDU), and HIV-positive men who have unprotected sex with men (MSM) have a high prevalence of HIV/HCV co-infection^2^. Co-infection results in an accelerated course of chronic hepatitis and rapid progression of liver disease to terminal stages of cirrhosis and hepatocellular carcinoma (HCC)^3,4^. In addition, HIV/HCV infection is associated with increased cardiovascular morbidity, renal dysfunction and various types of cancer other than HCC^5,6^. All these factors may reduce overall survival in HCV co-infected patients, beyond that of HIV-mono- infected patients.

The introduction of combined antiretroviral treatment (cART) has dramatically improved mortality from opportunistic diseases and AIDS in HIV positive patients^7,8^. Thus, morbidity and mortality from HCV co-infection has gained increased importance, and liver-related mortality remains a crucial problem in co-infected patients^9^. HIV/HCV-positive patients are more likely to experience liver toxicity from cART^8^. HIV/HCV co-infected patients show reduced CD4-cell recovery on cART and may suffer greater damage from immune activation due to HIV replication than patients with HIV infection alone^10,11^. Thus, HCV infection constitutes an independent risk factor for all-cause mortality in HIV-infected patients^12,13^.

On the other hand, cART exerts protective effects and improves overall and liver- related mortality in HIV/HCV co-infected patients^14^. Early cART administration mitigates liver fibrosis progression in HIV infected patients and thus is expected to markedly reduce mortality in co-infected patients^15,16^. Likewise, cure of HCV infection by antiviral treatment has been reported to improve survival of HIV/HCV co-infected patients^17^. Achieving a sustained virologic response (SVR) after HCV therapy has been shown to reduce 5-year mortality rates both in HCV mono-infected and HIV/HCV co-infected patients as compared to those without SVR^18^.

Nevertheless these studies may suffer from selection bias, both in mono- and co-infected patients. Beyond that, positive effects of SVR on survival in HIV/HCV co-infected patients were not confirmed by all studies, probably reflecting that some patients with advanced cirrhosis may have had less benefit, because liver fibrosis and impaired liver function persisted after HCV cure^19^.

The Bonn HIV cohort prospectively follows patients with both HIV- and HIV/HCV infection at regular intervals and provides an excellent longterm data base to study regional risk factors contributing to mortality of HIV infection in the various phases of the HIV pandemic. Here, we report survival in Caucasian patients with HIV/HCV co-infection between 2004, when cART had been fully established and 2014, when the first new generation direct acting antivirals (DAAs) were approved in Germany.

## Patients and Methods

### Patients

In 2002 we initiated a longitudinal survival study in the Caucasian patients of the Bonn HIV cohort. We included patients with HIV infection with or without concomitant HCV infection, who presented at our out-patient department between January 1^st^ 2004 and January 1^st^ 2014 and had sufficient follow-up data. We excluded 88 patients (43 HIV/HCV co-infected and 45 HIV mono-infected patients), because they did not have any follow-up visit, and 16 patients due to missing longitudinal data. In order to minimize bias from ethnic diversity 51 further patients (13 HIV/HCV co-infected and 38 HIV mono-infected patients) were excluded because they had African descent. All remaining patients were Caucasians and had regular visits every 3 months. We recorded any complications and classified them according to the European modification of the Centers for Disease Control and Prevention criteria.

Here, we analyzed all-cause-mortality by the Kaplan-Meier method between January 1^st^ 2004 and January 1^st^ 2014, and identified independent predictors of mortality by Cox’s regression analysis over the whole observation period.

HIV-positive patients received cART following the regularly updated guidelines for antiretroviral treatment as recommended by the European AIDS Clinical Society (EACS). Patients started their treatment at different timepoints as treatment indications were individually different for each patient, therefore observation period was restricted to a defined 10 year period of time. In patients with HIV/HCV co-infection, we offered a combination therapy with pegylated-interferon with or without ribavirin unless contraindications were present.

Blood counts and liver-function tests (alanine and aspartate aminotransferases, alkaline phosphatase, bilirubin, and y-glutamyltranspeptidase) were determined by routine biochemical procedures. Serum and EDTA blood were drawn to determine HIV viral loads. CD4-positive T cells were counted every 3 months on a FACS flow cytometer according to the manufacturer’s instructions (Becton Dickinson, Heidelberg, Germany).

All patient data were regularly documented in the “Medeora” electronic surveillance system and extracted from this database for this analysis. None of the patients had significant alcohol intake (>30 g alcohol/day). Tabacco use was not documented in the electronic surveillance system.

### Viral diagnostics

In all subjects initial HIV serology was tested with a commercially available HIV antibody ELISA and p24 antigen assay (Abbott, Wiesbaden; Coulter, Hamburg, Germany) according to the manufacturers’ instructions. HIV loads were determined quantitatively using the NucliSens HIV-QT assay (Organon Teknika, Boxtel, the Netherlands). Amplified patient RNA was quantified with different electrochemiluminescent probes in the NASBA QR system (Organon Teknika, Boxtel, the Netherlands). This assay had a detection limit of 80 copies/ml.

All patients were tested for HBsAg, anti-HBs, and anti-HBc with commercial assays (Abbott, Wiesbaden, Germany). HCV RNA was isolated from serum samples and HCV RNA checked annually. HCV genotypes were determined by the Innolipa II line probe assay (Innogenetics, Zwijndrecht, Belgium) according to the manufacturer’s instructions. After 2007, HIV and HCV viral loads were quantified by real time polymerase chain reaction using the Abbott RealTime m2000rt (Abbott Laboratories, Illinois, USA) with detection limits of 15 U/ml for HCV and 40 copies/ml for HIV.

HIV viral load was documented at study recruitment (2002–2003), 2004, 2008 and at the end of observation/2014. Patients were classified as complete responders (CR), when their HIV RNA on cART was detectable (detection limit 40 copies/ml) on no more than two timepoints within the observation period. All other patients with detectable HIV viral load on more than two occasions were considered to represent incomplete responders (IR).

### Statistical analysis

Patients were stratified into the two groups HIV mono-infected (HIV) versus HIV/HCV co-infected patients (HIV/HCV). Differences in demographic, viral and biochemical parameters were compared between the groups by Student’s *t* test, chi-2 test, and the non-parametric Mann-Whitney test, as appropriate.

We analyzed overall survival in each group using the Kaplan-Meier method and a logrank test. Likewise we compared survival between CR and IR separately in HIV− and HIV/HCV-infected patients. Survival was assessed from 01.01.2004 until 01.01.2014 or until death or last patient visit. Ten patients without cART treatment were excluded from the analysis. All surviving patients were censored on the 01.01.2014 or last visit. This observation period was chosen, in order to cover the period before introduction of new DAAs against HCV, which in Germany obtained first approval as interferon-free therapeutic options in 2014.

Causes of death were checked independently (J.K.R) and recorded. For the purpose of survival analysis patients with liver transplantation were considered to represent “death from liver failure” taking the date of transplantation as a surrogate date of death.

Survival was studied by a Cox’s regression model in order to identify factors independently associated with long-term mortality. Potential confounders for the initial analysis included clinical features (sex, age, presence of hepatitis B and C, respectively, class of antiretroviral drugs, anti-HCV therapy and its outcomes) as well as laboratory results such as HCV and HIV loads, alanine amino transferase, aspartate aminotransferase, y-glutamyltranspeptidase, bilirubin and CD4 T cell counts. Variables at study entry were compared by univariate analysis (ANOVA) and entered stepwise into a forward conditional regression model, if a difference with p < 0.1 was detected between dead and surviving patients. The conditions for inclusion and exclusion of variables in the final model were p < 0.05 and p > 0.10, respectively. All statistical analyses were performed using the SPSS software package (version 22).

### Compliance with ethical standards

The study was approved by the local ethics committee of the University of Bonn and all procedures performed in this study involving human participants were in agreement with the 1975 Declaration of Helsinki. Written informed consent was obtained from the patients prior to enrollment.

## Results

### Patient characteristics

Our cohort initially comprised 487 patients, who had either HIV mono-infection (n = 269) or HIV/HCV-infection (n = 218). To minimize bias resulting from ethnic diversity, we restricted our analysis to Caucasian patients and also excluded 104 patients with incomplete longitudinal data or missing follow up... as well as 10 patients without cART. The remaining 322 patients constitute the study cohort of this report, consisting of 176 patients with HIV infection, and 146 patients with HIV/HCV co-infection. Demographic and clinical data for each patient group are summarized in Table 1. Patient disposition has been summarized in Supplementary Table 1.

**Table 1:** Patient characteristics.

	HIV	HIV/HCV	p-value
**Total number of patients (%)**	**176 (54**.**7)**	**146 (45**.**3)**	—
**Demographic data** Age [median years, range]	43 (20–76)	41 (24–65)	0.007
Gender: male/female [% male]	153/23	129/17	0.699
**Risk category** n [%]			
Haemophilia Intravenous drug use Homosexual Heterosexual Unknown routes of transmission	4 (2.3) 3 (1.7) 98 (55.7) 27 (15.3) 44 (25.0)	88 (60.3) 37 (25.3) 9 (6.2) 5 (3.4) 7 (4.8)	<0.001 0.001 <0.001 0.001 0.001
**CD4 T-cell counts at study entry** [cells/μl]	423 (8–1303)	372 (6–1941)	<0.001
**CD4 T-cell counts at end of study** [cells/μl]	530 (35–1800)	361 (5–1765)	<0.001
**Platelets [G/L]**	205 (64–395)	174 (30–435)	0.001
**AST/GOT [U/L]**	26 (4–168)	38 (7–536)	0.004
**ALT/GPT [U/L]**	29 (11–122)	55 (12–449)	0.002
**GGT [U/L]**	39 (3–669)	64 (7–957)	0.007
**Bilirubin [mg/dl]**	0.49 (0.18–3.36)	0.64 (0.15–7.23)	0.029
**Hepatitis B serology** **HBs Ag positive n (%)**	17 (9.7)	30 (20.6)	0.018
**HCV treatment (%)** **SVR (%)**	—	56 (38.4) 30 (20.6)	n.a.
**HIV loads** **at study entry** [median copies/ml, range] **at last observation**	0 (0.0–456248) 0 (0.0–456248)	0 (0.0–283990) 0 (0.0–587508)	0.805 0.179
**Patients with detectable HIV VL n [%**]			
At study entry At end of observation/death	75 (42.6) 26 (14.8)	71 (48.6) 22 (15.1)	0.293 0.883
**HAART regimes** n [%]			
NRTI NNRTI PI Other antiretrovirals	17 (9.7) 27 (15.3) 117 (66.5) 3 (1.7)	16 (10.9) 19 (13.0) 92 (63.0) 4 (2.7)	0.869 0.653 0.615 0.465

ALT: alanine aminotransferase; AST: aspartate aminotransferase; GGT: γ–glutamyl transferase; HCV: hepatitis C virus; HBs Ag: hepatitis B virus s Antigen.

NRTI: Nucleoside Reverse Transcriptase Inhibitors; NNRTI: Non Nucleoside Reverse Transcriptase Inhibitors; PI: Protease inhibitors.

n.a.: not applicable.

Some differences were noted between our HIV and HIV/HCV co-infected patients: Co-infected patients were younger than HIV mono-infected patients (p = 0.007) and hemophilia was their leading risk factor (60.3%, p = <0.001), while HIV mono-infected patients were predominantly MSM (55.7%, p = <0.001).

As expected, patients with HIV/HCV infection had significantly higher liver enzymes (AST p = 0.004, ALT p = 0.002 and GGT p = 0.007), while their platelet counts (p = 0.001) were lower than in patients with HIV infection alone. Presence of liver cirrhosis was not consistently evaluated within the cohort and therefore not considered for the statistical analysis.

HIV/HCV infected patients had also less efficient reconstitution of their CD4 counts and their average CD4 counts at last observation were significantly lower than those of HIV-mono-infected patients (361 vs. 530; p = <0.001).

### Infection control by cART and HCV treatment

At entry into the observation period (01.01.2004) 270 (83.9%) patients were already on cART and 146 (45.3%) patients had detectable HIV viral loads (HIV 42.6%, HIV/HCV 48.6%). However, median HIV viral loads did not differ between HIV and HIV/HCV infected patients. In the remaining patients cART was initiated during the observation period.

In the majority of patients a protease inhibitor-based regime was given (Table 1). Substances used in the antiviral regimes are listed in Supplementary Table 2.

Forty-eight patients (14.9%) achieved only incomplete antiretroviral response (HIV 14.7%, HIV/HCV 15%) while all other patients were complete responders.

### Analysis of survival

The average observation periods were comparable for both patient groups (HIV 3047 days vs. 3147 days), and at start of observation already treated patients had been on cART for a similar length of time (HIV 1902 days vs. HIV/HCV 1978 days).

Overall 46 (14.3%) patients died during the observation period (HIV infection: n = 23 (13.1%), HIV/HCV co-infection: n = 23 (15.8%). Causes of death are summarized in Table 2. Infections (n = 6, thereof 4 non-AIDS related and 2 AIDS related) and cardiovascular disease (n = 6) were the predominant causes of mortality in HIV-mono-infected patients.

**Table 2:** Causes of death in the Bonn cohort.

Number of deaths	HIV 23 (13.1%)	HIV/HCV 23 (15.8%)
**Causes of death**		
Infections/sepsis	4^#^ (17.4%)	3 (13.0%)
Cardiovascular diseases	6 (26.1%)	3 (13.0%)
Non-AIDS-Malignancies	—	1 (4.3%)
Lung cancer	2 (8.7%)	1 (4.3%)
Liver related deaths	1 (4.3%)	8 (34.8%) *p* = *0*.*03*
Liver failure	1 (4.3%)	2 (8.7%)
OLTX	—	2 (8.7%)
HCC	—	4 (14.4%)
Suicide/accidents	2* (8.7%)	
Intoxication	2 (8.7%)	1 (4.3%)
AIDS defining conditions	3 (13.0%)	3 (13.0%)
Pneumocytis jeroveci pneumonia	2 (8.7%)	2 (8.7%)
Non-Hodgkin lymphoma	—	1 (4.3%)
Castleman’s disease	1 (4.3%)	—
Cryptosporidiasis	—	—
Other causes	—	2 (8.7%)
Renal failure	—	1 (4.3%)
Bleeding due to Osler’s disease	—	1 (4.3%)
Unknown causes of death	3 (13.0%)	1 (4.3%)

*One patient committed suicide after a diagnosis of lung cancer.

^#^One patient died from combined MRSA sepsis and liver failure; a second patient had lung cancer before death from sepsis.

OLTX: liver transplantation, HCC: hepatocellular carcinoma.

In patients with HIV/HCV co-infection liver related deaths (n = 8, thereof 2 liver transplantations), infections (n = 5) and cardiovascular diseases (n = 3) were the major causes of death. Liver related mortality was significantly higher in HIV/HCV co-infected patients (8 vs. 1, p = 0.03). Only 6 deaths occurred from AIDS-defining conditions, and 4 deaths had to be attributed to malignancies unrelated to AIDS (thereof 3 patients with lung cancer).

**Figure 1:**
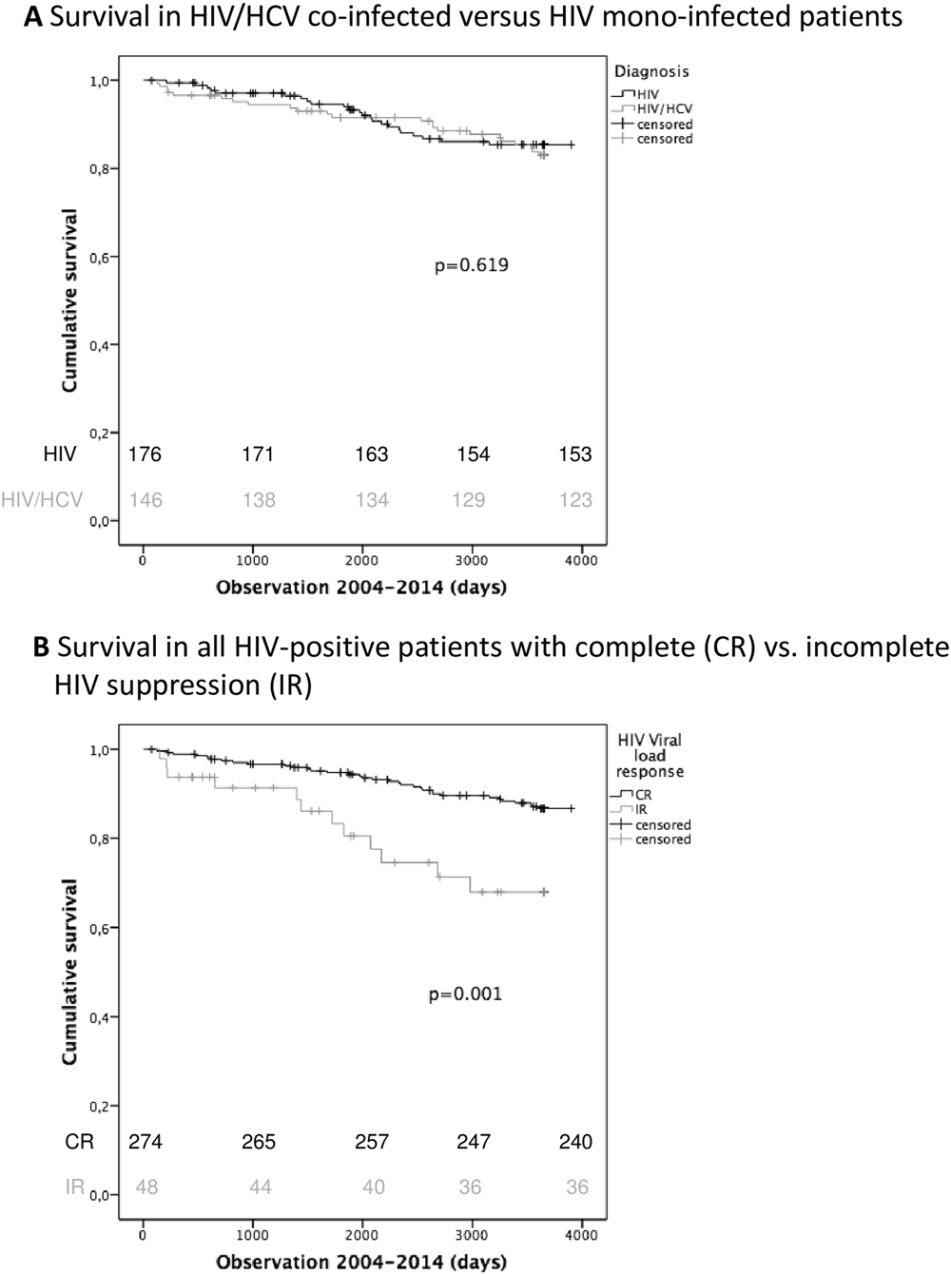
All-Cause Survival Analysis of HIV and HIV/HCV -infected patients under cART. (**A**) Kaplan-Meier plots comparing survival (in days) in HIV/HCV co-infected patients (grey line) to patients with HIV mono-infection (black line). Vertical marks indicate censored patients. The Log-rank test was used to test statistical significances. Survival does not significantly differ between HIV− and HIV/HCV infected patients (p = 0.619). (**B**) Kaplan-Meier plots comparing survival in all patients (HIV+ and HIV/HCV+) with respect to their quality of HIV control during the observation period. Patients with CR (black line) show significantly better survival than patients with IR (grey line) (p = 0.001).

Survival of patients groups was calculated by Kaplan-Meier analysis, with survival calculation starting from 01.01.2004. Interestingly survival analysis by the Kaplan-Meier method revealed only a minimal difference in survival between patients with HIV/HCV co-infection and HIV infection (p = 0.619) (Fig. 1A). To check, if treatment intensity might have affected survival, we re-calculated Kaplan-Meier analysis, comparing survival between patients with CR and IR as defined in the methods section. As expected, patients with IR had significantly higher mortality rates (p = 0.001) as compared to patients with CR (Fig. 1B).

**Figure 2:**
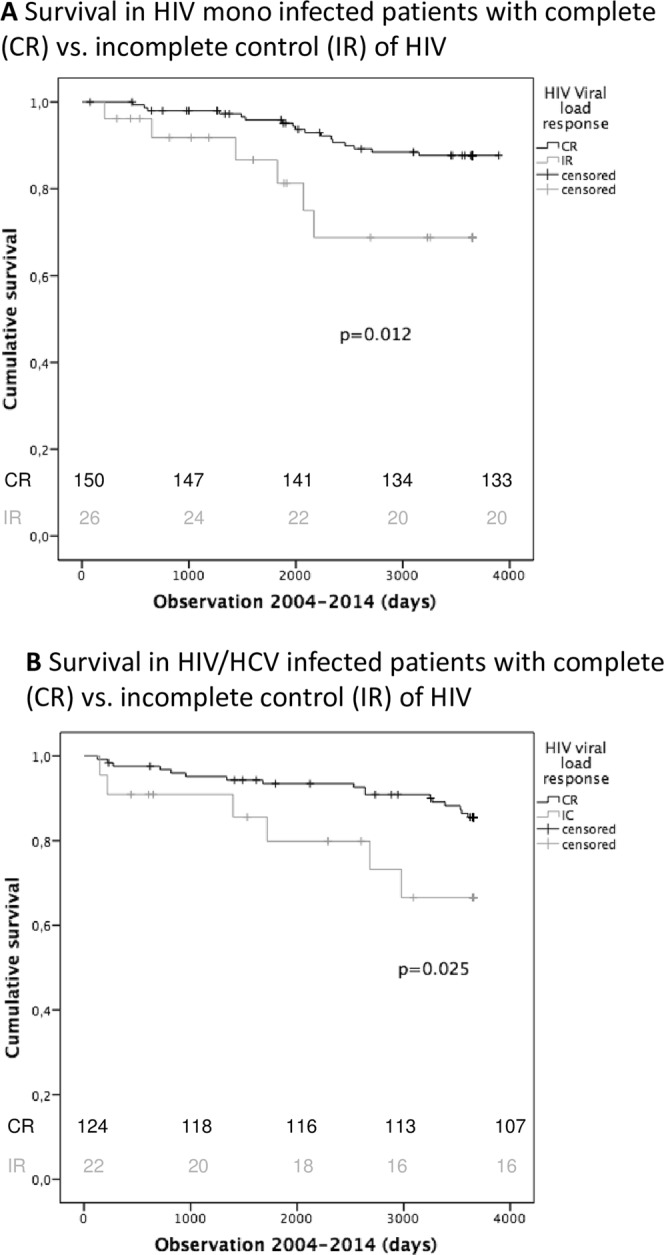
Kaplan-Meier Plots comparing survival of HIV mono- and HIV/ HCV co-infected patients with respect to their CR status. (**A**) Kaplan-Meier plots comparing survival (in days) in HIV infected patients with CR (black line) versus IR (grey line). Vertical marks indicate censored patients. The Log-rank test was used to test statistical significances. Survival differs significantly differ between patients with CR and IR (p = 0.012). (**B**) Kaplan-Meier plots comparing survival in HIV/HCV co-infected patients with respect to their quality of HIV control during the observation period. Patients with CR (black line) show significantly better survival than patients with IR (grey line) (p = 0.025).

This benefit of CR was seen independently both in HIV (HIV p = 0.012; 3652 days vs. 2986 days) and HIV/HCV positive patients (HIV/HCV p = 0.025; 3426 vs. 2997 days), see Fig. 2.

A Cox regression model was calculated for all patients as described in the methods section. Table 3 summarizes the results: Overall Cox regression identified age (p = 0.008), y-glutamyltranspeptidase (p < 0.001) and bilirubin (p = 0.008) as independent predictors (Table 3) of survival.

**Table 3:** Independent risk factors for all-cause-mortality in HIV and HIV/HCV positive patients.

Parameter	OR	95%-CI	p-value
Univariate analysis			
HIV VL at study entry [copies/ml]	0.346	0.178–0.670	***0***.***002***
HIV VL at end of observation [copies/ml]	0.425	0.202–0.896	***0***.***024***
CD4 (end of observation) [cells/μl]	0.997	0.995–0.998	<***0***.***001***
CD4 (at start of observation) [cells/μl]	0.997	0.996–0.999	***0***.***002***
AST/GOT [U/ml]	1.009	1.002–1.017	***0***.***016***
GGT [U/ml]	1.007	1.003–1.011	<***0***.***001***
Bilirubin [mg/dl]	1.807	1.186–2.753	***0***.***006***
Age [years]	1.041	1.010–1.074	***0***.***010***
Diagnose (HIV vs. HIV/HCV)	1.076	0.806–1.437	0.620
Outcome HCV therapy	0.827	0.346–2.335	0.827
Transmission Haemophilia	1.155	0.604–2.207	0.664
Transmission MSM	0.924	0.499–1.712	0.802
Delay in start of cART	1.000	1.000–1.000	0.738
Multivariate analysis*			
Bilirubin [mg/dl]	1.789	1.165–2.748	0.008
GGT [U/ml]	1.003	1.002–1.005	<0.001
Age [years]	1.042	1.011–1.074	0.008

*Including all significant parameters from the univariate analysis.

GGT: Gamma-glutamyltranspeptidase, AST: Aspartate Aminotransferase.

In order to assess the potential bias resulting from HIV infection before beginning cART, we recalculated our Cox regression model entering the delay between time of first HIV diagnosis and start of cART as additional covariate. Although, cART had been initiated with a significantly greater delay in HIV/HCV co-infection than in patients with HIV mono-infection (HIV: median 592 days vs. HIV/HCV median 4052 days; p = 0.0001), this calculation failed to reveal any major effect of time before cART on survival in 2004 to 2014 (p = 0.261). Next we evaluated the effect of viral control at start of observation (01.01.2004) on survival. To this end we re-calculated Kaplan-Meier survival with respect to viral loads at start of observation separately in HIV and HIV/HCV patients (Supplementary Fig. 1). Survival was significantly better in those patients who already had undetectable HIV viral loads at start of observation (p = 0.001 for all patients). This effect was markedly greater in HIV/HCV-positive patients (Fig. 2B; p = 0.006; 35266 days vs. 3194 days) than in HIV-mono-infected patients (Fig. 2A; p = 0.075; 3670 days vs. 3287 days).

Finally, we also studied the potential impact of HCV infection or interferon-based HCV antiviral treatment on all-cause mortality by Kaplan-Meier analysis. Fifty-six of the 146 co-infected patients (38.4%) received anti-HCV combination therapy with interferon ± ribavirin, which led to a sustained virologic response (SVR) in 30 (20.6%) of the treated patients. Neither HCV antiviral treatment nor achieving a SVR under therapy significantly modified survival (Supplementary Fig. 2). This finding is further supported by the fact that Cox regression could identify neither “presence of HCV infection” nor “HCV treatment” and “SVR” as significant factor determining survival in our patients.

## Discussion

Mortality from opportunistic diseases has substantially decreased in HIV infected patients after antiretroviral combination therapy became available^20^. It has even been proposed that life expectancy on cART might approach that of uninfected populations, when HIV-positive patients receive optimum treatment^21,22^. As deaths from AIDS have declined, liver diseases have emerged as leading cause of mortality in HIV infected patients^23,24^. Thus, chronic hepatitis C and its complications (cirrhosis, liver cancer) pose a particularly severe problem when additional HCV infection is present in HIV-positive patients^25,26^. However, the majority of epidemiological data on HIV/HCV co-infection have been reported before cART was established or stem from publications on patient groups with limited access or treatment adherence. Most reports about the survival of HIV/HCV co-infected patients are based on mainly IVDU patients and report a reduced survival in co-infected patients^27–29^. However, follow up in such cohorts is shorter and more affected by life style factors than in our cohort, where we had regular contacts with HIV- and HIV/HCV-co-infected patients over the 10 years 2004 to 2014. We assessed the effects of HCV co-infection in HIV-positive patients who had liberal access to antiretroviral drugs during the period before HCV-specific DAAs had become available. Although death owing to liver disease was significantly more frequent in the HIV/HCV co-infected group, overall survival differed only marginally between HIV-mono-infected and HIV/HCV co-infected patients. This conclusion is derived from the fact, that Kaplan-Meier survival curves were not significantly different between HIV and HIV/HCV patients. Furthermore, Cox regression analysis failed to identify any HCV-related parameter as predictor of mortality. This difference to other studies in HIV positive patients^12,13^ is likely to reflect differences between the HIV/HCV-positive study groups. While HIV/HCV co-infected patients in the other studies largely comprised individuals with IVDU^27^, our HIV/HCV group consisted mostly of hemophiliac patients who show high adherence and are less prone to bias from unfavorable life style factors. On the other hand, hepatitis and liver disease in patients with IVDU are also likely to reflect additional liver damage owing to hepatic drug toxicity. Cox regression analysis in our patients identified age, yGT and bilirubin as predictors of survival irrespective from HCV co-infection. This is in line with previously established surrogate markers for advanced liver damage in HIV-positive patients^4^.

Finally, part of our HCV/HIV co-infected patients quite likely had already advanced liver disease, because elevated liver enzymes and reduced platelet counts indicated impaired liver function and portal hypertension at study begin. Then, liver damage cannot be sufficiently restored by SVR and an increased mortality risk persists as described in other cohorts of HIV/HCV co-infected hemophiliac patients^19^. This could explain, why liver related mortality still constitutes a major cause of death in our patients.

Finding rather similar survival in the HIV/HCV co-infected and HIV mono-infected patients of our cohort was an unexpected observation. Two conclusions are possible from this observation: First, risks in HIV and HIV/HCV co-infection seem to compete for the same subgroup of patients so that a rather similar pool of patients will die from any other cause who otherwise might have died from liver disease if HCV co-infection had been present. In line with this hypothesis, liver-related mortality remained a major cause of death in HIV/HCV co-infected patients despite improved overall survival on cART. Conversely, cardiovascular events constituted the main causes of death in HIV mono-infected patients. A second conclusion is that especially HIV/HCV-infected patients seem to have an increased benefit from cART, which almost completely compensates for negative effects of untreated HIV/HCV co-infection on survival. Of note, a more detailed survival analysis in our cohort suggests that primarily the quality of HIV inhibition determined chances of survival in either group of HIV-infected patients. Irrespective from HCV, patients who achieved CR over HIV replication had significantly better survival than those who occasionally revealed some detectable HIV RNA in their serum. This observation is strengthen by the observation that patients, who had initiated cART before and whose serum HIV RNA was below the level of detection at start of observation, had markedly better survival than those with detectable HIV both in the HIV-mono-infected and the HIV/HCV-co-infected subgroups of our cohort. This finding provides circumstantial evidence that antiretroviral therapy leads indeed to considerably greater survival benefits in patients with HIV/HCV co-infection. Thus, the current study confirms previous observations from a 12-year observational study of the pre-cART era that reported improved survival depending on the quality of antiretroviral therapy^14^, and extends this concept to patients living in the cART era. Beyond that, cART can stop progression towards liver fibrosis and reduce hepatic necro-inflammatory activity^30,31^.

Interestingly, our analyses did not reveal any impact of anti-HCV therapy on survival in our HIV/HCV-infected patients, while some previous publications had reported positive effects of HCV treatment and achieving a SVR on patient survival^16,17^. However, the number of our patients, in whom HCV therapy was attempted, was rather low and yielded only moderate outcomes, because similar to other reports the only available treatment option, prolonged interferon/ribavirin combination treatment, was accepted in our patients only infrequently^32^. Furthermore, survival in the EuroSIDA cohort was not influenced by the level of HCV viremia, and other studies equally failed to confirm a positive impact of SVR on survival^19,33^.

Additionally, co-infected patients had a lesser increase in CD4 counts, which might reflect advanced portal hypertension. CD4 nadir was lower in co-infected patients at study entry, which could explain the overall lower CD4 reconstitution.

Further more, cART war initiated with delay in co-infected patients. Unfortunately, there was no data available on whether time since HCV diagnosis had an impact on survival, as the date of HCV diagnosis was not documented.

Our study is based on the analysis of a prospectively followed patient cohort, which collected observational data but did not control for treatment allocation. While this is probably the best type of study which can be done realistically in HIV-positive patients, because it reflects daily practice, this study type is prone to potential bias. Treatment adherence is certainly a critical source of bias, which was apparently exceptionally good in our cohort. This might explain, why less survival benefit has been reported for HIV/HCV-infected patients in other patients populations with more variable patient adherence, e.g. in the US Veterans Administration Study^34^. Further bias may result from the fact that several patients in our cohort had survived the pre-cART era, which may inadvertently selected for the fittest. To address this issue, we analyzed if the individual length of the period between first diagnosis of HIV infection and start of cART was associated with differences in survival. It was re-assuring that we could not detect such effects. Thus, together with the finding, that other adverse factors at study entry such as lower CD4 counts in the HIV/HCV co-infected patients, did also not correspond to differences in survival on cART makes us confident that indeed the quality of HIV control is the primary key factor of survival irrespective from HCV co-infection.

Taken together our results support the concept that early start of cART together with optimum patient care and surveillance in order to ensure continued complete suppression of HIV replication below the level of detection is of pivotal importance for achieving survival rates of HIV/HCV co-infected patients similar to those of HIV mono-infected patients. The availability of new DAAs enabling interferon-free HCV therapy offers additional chances for HIV/HCV co-infected patients^16^ and it is challenging to predict, that broad use of this new option might improve survival in well controlled HIV/HCV co-infected patients to the level of an uninfected population.

## Supplementary information

Supplementary information
