# Supplementary material for: Survival of HIV/HCV co-infected patients before introduction of HCV direct acting antivirals (DAA)
Source: Sci Rep. 2019 Aug 29;9:12502. doi: 10.1038/s41598-019-48756-3 (PMC6715635; doi:10.1038/s41598-019-48756-3)
Supplement: Supplementary file 1 — Supplementary information [file 41598_2019_48756_MOESM1_ESM.pdf]

Survival of HIV/HCV co-infected patients before introduction of HCV direct acting antivirals (DAA)

L Dold, C Schwarze-Zander, C Boesecke, R Mohr,
B Langhans, J-C Wasmuth, CP Strassburg, JK Rockstroh,
U Spengler

A Survival in HIV mono infected patients with undetectable vs. detectable HIV viral load at start of observation

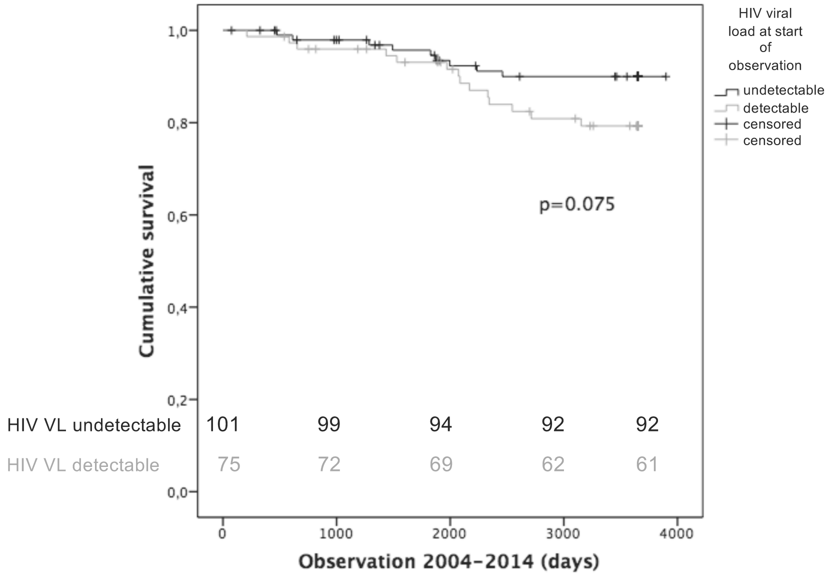

B Survival in HIV/HCV infected patients with undetectable vs. detectable HIV viral load at start of observation

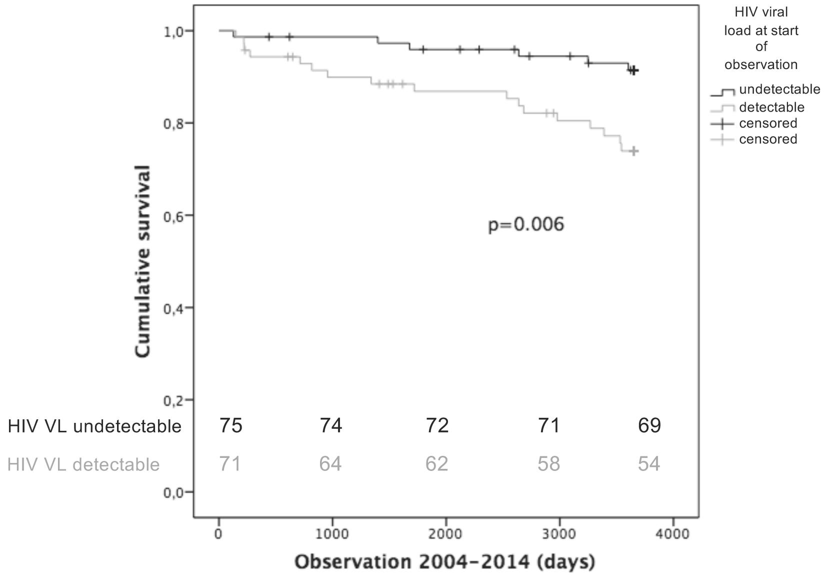

Supplementary Figure 1: Kaplan-Meier Plots comparing survival of HIV mono- and HIV/ HCV co-infected patients. Kaplan-Meier plots comparing survival in the HIV mono-infected patients between patients with undetectable HIV viral loads at study entry (black line) versus patients with detectable HIV viral load at study entry (grey line). Vertical marks indicate censored patients. Survival [days] was calculated from 01.01.2004. The difference in survival between the groups was not significant but showed a trend for better survival in those patients with undetectable HIV viral load at start of observation (Log-rank test: p=0.075). Kaplan-Meier plots comparing survival in the HIV/HCV co-infected patients between patients with undetectable HIV viral loads at start of observation (black line) versus patients with detectable HIV viral loads at start of observation (grey line). Vertical marks indicate censored patients. Survival [days] was calculated from 01.01.2004. The difference in survival between the groups was significant (Log-rank test: p=0.006). The gain in survival was greater in HIV/HCV + patients with undetectable HIV RNA than of patients than in the patients with HIV+ patients.

A Survival analysis in HCV treated vs. untreated HIV/HCV positive patients

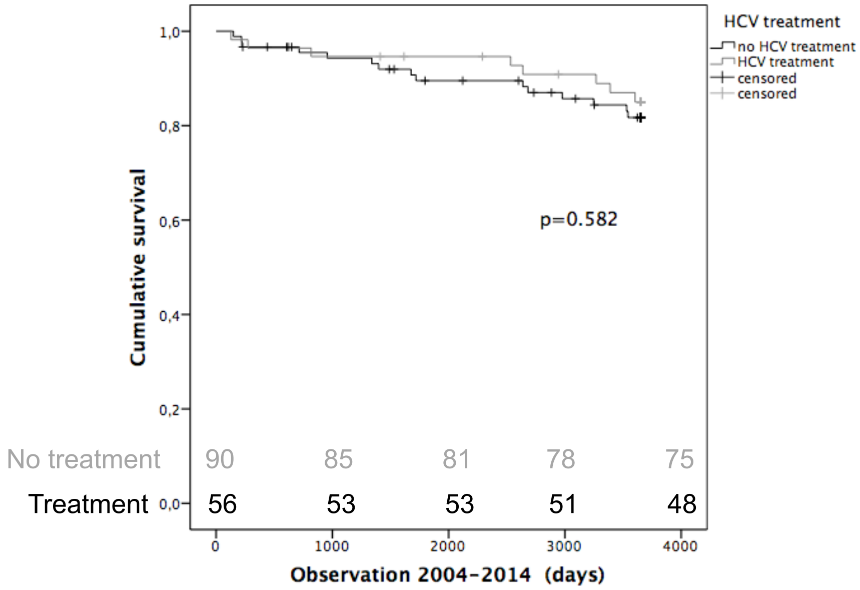

B Survival analysis in HIV/HCV patients who received HCV treatment, depending on HCV treatment outcome

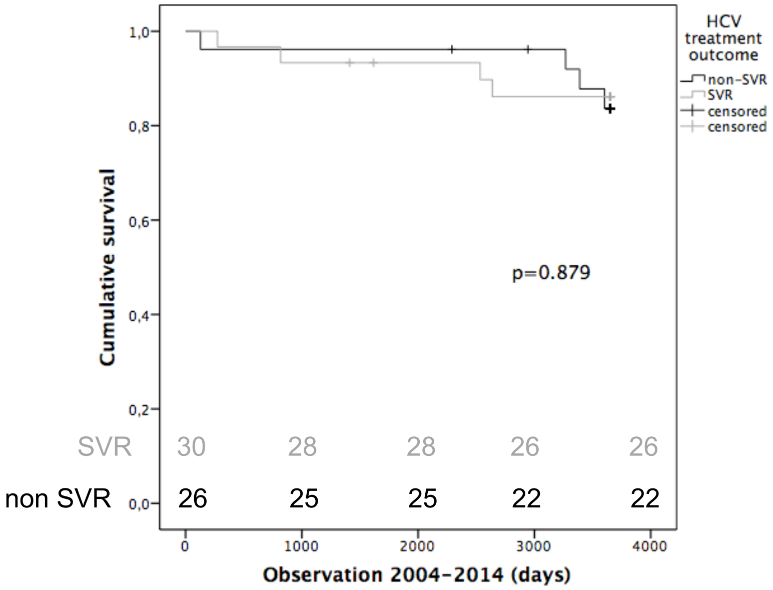

Supplementary Figure 2: Kaplan-Meier Plot analyzing effects HCV-treatment on survival of HIV/HCV co-infected patients. Kaplan-Meier plots comparing survival of co-infected patients who received interferon based anti HCV treatment (black line) to patients with no treatment (grey line). Vertical marks indicate censored patients. Survival was compared statistically by a log rank test and did not significantly differ between the patient groups (p=0.582; 3327 vs. 3425 days).
Kaplan-Meier plots of survival curves in the 56 HIV/HCV co-infected patients who had received HCV antiviral treatment comparing patients who achieved sustained virologic response (SVR) (grey line) and treatment failures (black line). Survival curves were compared statistically by a log rank test and did not indicate any significant difference (p=0.879; 3488 vs. 3369 days).

Supplementary Table 1: Patient disposition

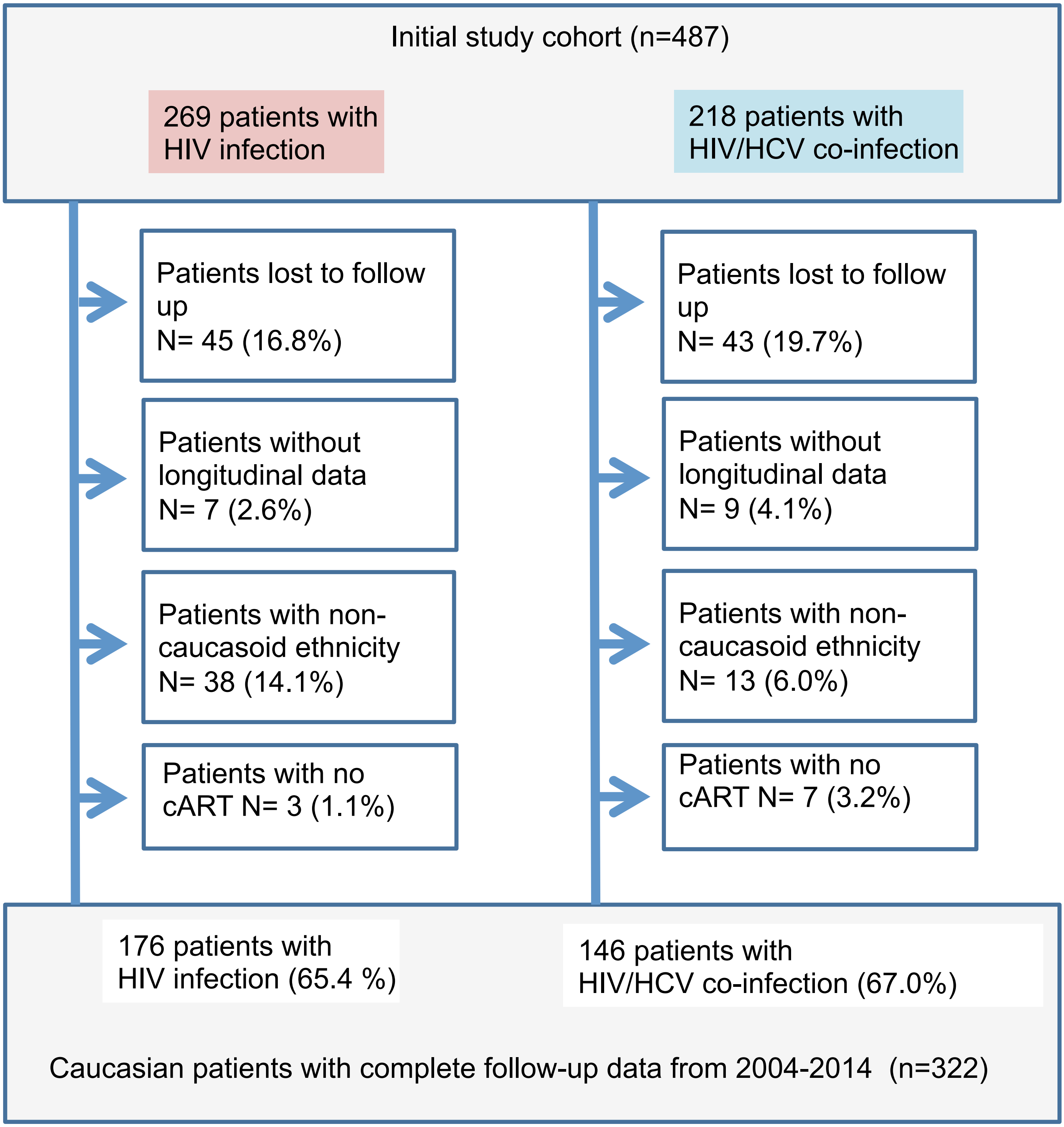

Supplementary Table 2: Antiviral substances

Antiviral substances	HIV	HIV/HCV
**Protease inhibitors (PI)**		
Saquinavir	16.5 %	11.0 %
Lopinavir	34.1 %	33.6 %
Indinavir	14.2 %	12.3 %
Atazanavir	5.1 %	6.8 %
Darunavir	0.6 %	4.1 %
Fosamprenavir	2.3 %	2.1 %
Nelfinavir	12.5 %	8.9 %
Tipranavir	1.2 %	------
Ritonavir (booster dose)	21.0 %	19.9 %
**Non Nucleoside Reverse Transcriptase Inhibitor (NNRTI)**		
Evavirenz	24.4 %	18.5 %
Nevirapin	11.4 %	6.2 %
**Nucleoside Reverse Transcriptase Inhibitors (NRTI)**		
Azidothymidine	43.8 %	42.5 %
Abacavir	36.4 %	19.9 %
Didanosine	13.1 %	13.0 %
Tenofovir	28.4 %	40.4 %
Lamivudine	77.8 %	64.4 %
Stavudine	51.1 %	30.8 %
**Entryinhibitors**		
Enfuvirtide	1.7 %	-----
Maraviroc	-----	0.8%
**Integraseinhibitors**		
Raltegravir	2.2 %	1.2 %
